# Identification of beneficial and detrimental bacteria impacting sorghum responses to drought using multi-scale and multi-system microbiome comparisons

**DOI:** 10.1038/s41396-022-01245-4

**Published:** 2022-05-06

**Authors:** Mingsheng Qi, Jeffrey C. Berry, Kira M. Veley, Lily O’Connor, Omri M. Finkel, Isai Salas-González, Molly Kuhs, Julietta Jupe, Emily Holcomb, Tijana Glavina del Rio, Cody Creech, Peng Liu, Susannah G. Tringe, Jeffery L. Dangl, Daniel P. Schachtman, Rebecca S. Bart

**Affiliations:** 1 Donald Danforth Plant Science Center, St. Louis, MO USA; 2 Washington University, St. Louis, MO USA; 3 Department of Biology, University of North Carolina at Chapel Hill, Chapel Hill, NC USA; 4 Howard Hughes Medical Institute, University of North Carolina at Chapel Hill, Chapel Hill, NC USA; 5 Curriculum in Bioinformatics and Computational Biology, University of North Carolina at Chapel Hill, Chapel Hill, NC USA; 6 DOE Joint Genome Institute, Lawrence Berkeley National Laboratory, Berkeley, CA USA; 7 Department of Agronomy and Horticulture, University of Nebraska-Lincoln, Lincoln, NE USA; 8 Department of Statistics, Iowa State University, Ames, IA USA; 9 Environmental Genomics and Systems Biology Division, Lawrence Berkeley National Laboratory, Berkeley, CA USA; 10 Carolina Center for Genome Sciences, University of North Carolina at Chapel Hill, Chapel Hill, NC USA; 11 Curriculum in Genetics and Molecular Biology, University of North Carolina at Chapel Hill, Chapel Hill, NC USA; 12 Department of Microbiology and Immunology, University of North Carolina at Chapel Hill, Chapel Hill, NC USA; 13 Center for Plant Science Innovation, University of Nebraska – Lincoln, Lincoln, NE USA; 14 Present Address: Department of Plant and Environmental Sciences, Institute of Life Science, The Hebrew University of Jerusalem, Jerusalem, Israel

**Keywords:** Plant sciences, Microbiome, Bacterial host response

## Abstract

Drought is a major abiotic stress limiting agricultural productivity. Previous field-level experiments have demonstrated that drought decreases microbiome diversity in the root and rhizosphere. How these changes ultimately affect plant health remains elusive. Toward this end, we combined reductionist, transitional and ecological approaches, applied to the staple cereal crop sorghum to identify key root-associated microbes that robustly affect drought-stressed plant phenotypes. Fifty-three *Arabidopsis*-associated bacteria were applied to sorghum seeds and their effect on root growth was monitored. Two *Arthrobacter* strains caused root growth inhibition (RGI) in *Arabidopsis* and sorghum. In the context of synthetic communities, *Variovorax* strains were able to protect plants from *Arthrobacter*-caused RGI. As a transitional system, high-throughput phenotyping was used to test the synthetic communities. During drought stress, plants colonized by *Arthrobacter* had reduced growth and leaf water content. Plants colonized by both *Arthrobacter* and *Variovorax* performed as well or better than control plants. In parallel, we performed a field trial wherein sorghum was evaluated across drought conditions. By incorporating data on soil properties into the microbiome analysis, we accounted for experimental noise with a novel method and were able to observe the negative correlation between the abundance of *Arthrobacter* and plant growth. Having validated this approach, we cross-referenced datasets from the high-throughput phenotyping and field experiments and report a list of bacteria with high confidence that positively associated with plant growth under drought stress. In conclusion, a three-tiered experimental system successfully spanned the lab-to-field gap and identified beneficial and deleterious bacterial strains for sorghum under drought.

## Introduction

Many factors influence overall plant health and productivity including varietal differences (*G*_P_), abiotic stresses (*E*) and the diverse collection of microbes (*G*_M_) that live intimately in and around plants [1–3]. The composition as well as the spatial and temporal dynamics of the plant microbiota are also influenced by environmental conditions and host factors [4–10] resulting in a tangled web of interactions (*PlantHealth* = *G*_P_ × *G*_M_ × *E*). Previous research aimed at untangling this web of interactions can be divided into two general approaches: field-based surveys and controlled system experiments.

In field-based surveys, next generation amplicon sequencing is used to directly quantify microbial constituents associated with plants, often across various abiotic stresses [10–12]. These experiments group microbes into taxonomic units, often at the family or genus level, and are useful for observing major community shifts/differences. For example, it is well documented that compared to bulk soil, the root and rhizosphere contain much less microbial diversity, suggesting that plant roots influence the composition of their microbiomes [7, 9, 13]. Similarly, previous studies have shown that drought decreases the diversity of microbes in the roots of 30 angiosperm plants and 18 grass crop species including sorghum [7, 9, 10]. Notably, in these studies, *Actinobacteria* strains were enriched in both bulk soil and even more enriched in roots. From this observation, it was hypothesized that these Gram-positive (monoderm) bacteria display inherent physiological adaptation to drought as well as a response to plant metabolic changes under drought. Furthermore, some studies suggest that when plant hosts suffer from abiotic/biotic stresses, they recruit specific microbes able to alleviate the stress, known as the “cry for help” hypothesis [14, 15].

Biological nitrogen fixation and improved nutrient uptake by the mutualistic symbioses between legumes and rhizobia and between cereals and mycorrhizae, respectively, are among the most well-characterized examples of plant growth-promoting microbial processes and have been successfully studied in both lab and field settings [16]. Reductionist experiments within controlled systems have been used to probe the specific function of many additional beneficial microbes. However, in general, translating microbe-derived plant growth promoting phenotypes from labs into complex agricultural settings remains a challenge. For example, while *Azospirillum brasilense* strains promoted vegetative growth of maize and wheat in the greenhouse, they had little impact on plant growth in the field [17]. Recent efforts have used microbial synthetic communities (SynComs) as a reductionist model for natural microbiota. SynComs have been used to decipher *in planta* processes that lead to plant-microbiota homeostasis and to understand the mechanisms underlying the microbiota’s effects on plant growth, nutrient uptake and disease resistance [14, 18–20]. Berendsen et al discovered three rhizosphere bacterial species that are specifically enriched upon *Arabidopsis* foliar defense activation by the downy mildew pathogen [14]. These three strains were able to function synergistically in the field soils and induce systemic resistance to downy mildew disease. Voges et al. observed that iron deficiency caused a compositional shift in *Arabidopsis* roots and this was linked to changes in root exudation [19]. Most recently, using top-down deconstruction of a large phylogenetically diverse bacterial culture collection, it was demonstrated that the bacterial genus *Variovorax* [20], a core rhizosphere member across plant species and geographic locations [9, 20, 21], was able to protect *Arabidopsis* root growth from diverse root growth inhibitory strains. *Variovorax* strains protect the host plant from manipulation by hormone-secreting microbes within the microbiome, suggesting chemical interference as a novel strategy that enhances plant resilience.

Drought is one of the most important abiotic stresses for crop plants and sorghum [*Sorghum bicolor* (L.) Moench] is one of the best-adapted cereal crops to water-limited environments [22]. Decades of breeding have resulted in elite sorghum varieties and hybrids with optimized drought tolerance traits (*G*_P_ × *E*) including waxy leaf surfaces, deep root systems and the ‘stay green’ trait [23, 24]. In contrast, interactions between the root-associated microbiome and drought (*G*_M_ × *E*) and between the plant and the root-associated microbiome (*G*_P_ × *G*_M_), are less well understood.

Here, we test bacterial SynComs that affects *Arabidopsis* root growth [20] to determine whether a similar microbe-dependent phenotype is observed on sorghum. We tested the SynComs in a sorghum germination assay and a sorghum phenotyping assay and found that *Arabidopsis*-protective *Variovorax* strains can also protect sorghum growth from drought and root growth inhibition (RGI) from various bacterial strains. In parallel, we performed a sorghum field trial with well-watered and drought conditions. Drought-responsive microbes were identified including an enrichment of *Actinobacteria*, consistent with previous findings. Additionally, sorghum-associated bacteria, both beneficial and deleterious, were discovered from the phenotyping assay and the field trial. Several bacteria were observed to have phenotypic effects in both systems and so become high-priority candidates for future study. All three datasets suggest that *Arthrobacter* strains impair sorghum growth, especially under drought stress. To our knowledge, this is the first example of reductionist and ecological approaches revealing convergent results on crop plant associated microbial interactions relevant for a specific host plant trait.

## Materials and methods

### Sorghum root growth assay using germination paper (Fig. 1, Fig. S1)

#### Bacterial cultures

Detailed description of the 53 bacterial strains used in this work (Table S1 and [18, 20, 25]). Six days before each experiment, bacteria were streaked on NYGA plates with cycloheximide (5 g/L bactopeptone, 3 g/L yeast extract, and 20 mL/L glycerol, with 15 g/L agar for solid medium, 100 mg/L cycloheximide) from glycerol stocks. Bacteria were grown at 30 °C. After 4 days of growth, bacterial strains were re-streaked on fresh NYGA plates with cycloheximide and returned to the incubator for an additional 48-h of growth. Bacteria were resuspended into autoclaved, distilled water (optical density at 600 nm (OD_600_) = 0.5). For the synthetic communities (SynComs), equal volumes of individual bacterial cultures (OD_600_ = 0.5) were combined in a larger volume such that the final inoculum was OD_600_ = 0.5 and this mixture was used to aliquot an equal volume for each replicate.

#### Plant inoculation, growth, imaging and analysis

*Sorghum bicolor* (L.) Moench BTx623 seeds were surface sterilized in an airtight desiccator with chlorine gas by mixing 40 mL bleach with 5 mL saturated hydrochloric acid for 3-h and then were soaked in 10 mL of sterile water (control) or bacterial inoculum (individual strains or SynComs), overnight at room temperature. The soaked seeds were placed in the seed pockets of the germination pouch (CYG-38LB, PhytoAB, San Jose, CA). The germination pouches were placed vertically in dark folders, hung in file crates and plants were grown under a 14-h light/10-h dark regime (except all dark for the first day) with temperatures of 30 °C day/25 °C night and 50% humidity. Sorghum roots were imaged four and seven days after planting (DAP), using a document scanner. Primary root length elongation was manually measured using ImageJ. Primary root lengths were compared using a Wilcoxon rank-sum test. To control for false discovery rate (FDR), *p* values were corrected using the method of Benjamini-Hochberg. Significance of differences between treatments are indicated with asterisks showing adjusted *p* values.

#### Quantification of the colonized bacteria

For the rhizosphere versus endosphere assays (Fig S1), seeds were treated as above except that bacteria were grown directly from glycerol stocks for 48 h on plates and then seeds were inoculated with a 1.5 × 10^8^ CFU/mL (OD_600_ = 0.031 for *Variovorax* and OD_600_ = 0.3 for *Arthrobacter*). Seven DAP, two centimeter long root tip sections from two seedlings were cut and resuspended in 200 μL of wash buffer (10 mM of MgCl_2_, 0.05% Silvet-L77) by vortexing at maximum speed for 5 min. The supernatant, representing the rhizosphere, was transferred to a new tube and bacterial populations were determined by counting colony forming units (CFUs) of serial dilutions. The root sections were then surface-sterilized with a bleach solution (1% bleach, 0.1% Triton-X 100) for 4 min, followed by one wash with 70% ethanol and three washes with wash buffer. Aliquots of the final washes were plated on NYGA plates to determine effectiveness of root surface sterilization. The surface sterilized root sections were transferred into clean 2 mL Safe-lock tubes (Eppendorf) with 2.4 mm stainless steel beads and 200 μL of wash buffer, and were homogenized with a TissueLyser II (Qiagen) at 30 Hz for 1 min. The crushed root tissue solutions, representing endosphere, were used for serial dilutions. Aliquots of the dilutions were spread on NYGA plates. The resulting colonies were counted after 2-day incubation at 30 °C and calculated as CFU per unit of plant root tissue.

### Sorghum growth assay using the Lemnatec high-throughput phenotyping platform (Figs. 2 and 3, Figs. S2–S4)

#### Bacterial culture and plant inoculation

The SynCom strains were prepared for inoculation as described above. Each surface-sterilized sorghum seed was sown 2 inches deep into autoclaved foam plugs (Oasis, Kent, OH). Each SynCom inoculum was adjusted to OD_600_ = 0.5 and 13 mL of the corresponding microbial inoculants was poured over the foam plugs.

#### Lemnatec plant growth conditions

Sorghum seeds, with the microbial inoculants, were germinated in a Conviron growth chamber set to a 16-h day cycle with temperatures of 32/22 °C and humidity of 60/40% at day and night, respectively. After 2 days growth, germinated plugs were then transplanted into pre-filled, steam-sterilized, small tree pots (3 × 3 × 8 in.) with a one-to-one blend of Metro mix 360 and turface (Hummert International, Earth City, Missouri), that was water-saturated prior to transplanting. Each pot was loaded onto the Bellwether Phenotyping Platform. Growth conditions on the platform were set on a 16-h cycle with temperatures of 32/22 °C and humidity of 60/40% at day and night, respectively. Lighting was supplied by metal halide and high-pressure sodium bulbs set to emit 400 μmol*m^−2^*s^−1^. Water was delivered to the plants once per day by adding water to match the target weight of the given treatment. Target weights of 1158 g for well-watered and 854 g for drought were determined using the Decagon Soil Moisture Sensor and taking readings of fully saturated and completely dry soil. Interpolated values of 80% and 25% capacity were computed and used for the two treatments, respectively. To ensure plant viability and initial consistency, during the first four days after transplanting all plants were given water to match the well-watered weight and were also given an additional volumetric watering of 40 mL once per day. On the fifth day after transplanting, drought treatments were enforced.

#### Image segmentation and feature extraction

Imaging began 2 DAP. Every plant was imaged from two sides (0° and 90°) each day, for both visible and near-infrared (NIR) cameras, totaling four images per plant per day. All images were processed using the Bellwether workflow found in PhenotyperCV (https://github.com/jberry47/ddpsc_phenotypercv). Each image was color-corrected using a previously described algorithm [26] and had the background removed by image subtraction. To obtain a mask, a pipeline was employed that consists of a combination of: eroding, dilating, thresholding, region of interest (ROI) selection, and logical operators. Using the final mask, morphological characteristics, hue histogram and NIR histogram were extracted and written to file. The set of morphological characteristics obtained were: area, hull area, solidity, perimeter, width, height, center of mass *x*-coordinate (cmx), center of mass *y*-coordinate (cmy), number of hull vertices (hull_vertices), center of bounding ellipse *x*-coordinate (ex), center of bounding ellipse *y*-coordinate (ey), length of bounding ellipse major axis (emajor), length of bounding ellipse minor axis (eminor), angle of bounding ellipse (angle), bounding ellipse eccentricity (eccen), bounding ellipse circularity (circ), bounding ellipse roundness (round), bounding ellipse aspect ratio (ar), fractal dimension (fd), color correction strength (det), and indicator for out of frame (oof). As part of the feature extraction of the images, the NIR histogram for each image was produced. Post-processing of the histogram was done by normalizing the distribution by the size of the plant and calculating the average gray level for each image was done using weighted-mean estimation.

#### Outlier detection

Identification and removal of outliers was performed using Cook’s distance on a linear model that only included the interaction term of treatment, microbe, and time following Berry et al. [26]. This process resulted in approximately 7% of the data, 2029 images, being removed from further analysis.

#### Shapes ANOVA

To assess the variability to the drought, microbe, and interaction terms on each of the phenotypes, a fully random effect model was performed using R package lme4. For each phenotype, the sum of squares associated with each term was extracted and normalized to the total variance of the model to obtain the amount of variance explained by each component. The Pearson correlation matrix of all 20 phenotypes for all plant images on the last day was calculated and visualized using the R package corrplot. We used the plant area to estimate the effect of spatial distribution in the phenotyping growth chamber [27]. To aid the data exploration and visualization of raw data from PhenotyperCV and PlantCV pipelines, a shiny app (http://shiny.danforthcenter.org/PhenoAnalyzer/) was created and the plots (Figs. 2a, b and S2a, b) can be reproduced, along with additional analyses, using the raw data in the Zenodo repository (10.5281/zenodo.5703837). Please see Supplemental File 1 for more detail.

#### Phenotyper final harvest

On DAP23 image data was rapidly analyzed to identify outliers (outside of the 95% confidence interval based on plant area). The high-throughput phenotyping assay was concluded on DAP25 and 10 plants were randomly selected for each treatment, avoiding identified outliers. Shoot fresh weights and root samples (root plus rhizosphere) for each selected plant were collected. Shoot dry weights were recorded after drying at 37 °C and humidity of 30% for one week. Shoot water contents were calculated as the differences between shoot fresh and dry weights for each plant.

#### DNA extraction

Four 1-inch-long root sections and the attached soil were collected together for each plant. 2 mL Eppendorf Safe-Lock Tubes containing the samples were stored at −80 °C with four 2.38-mm stainless steel beads until processing. Root and soil samples were pulverized using a TissueLyser II (Qiagen) with cold blocks cooled in liquid nitrogen (2 min grinding, 30 Hz, 4 times). The orientation of sample cassettes in the TissueLyser was rotated between two grindings. DNA extractions were carried out on ground root and soil samples using DNeasy PowerPlant Pro kit (Qiagen) following the manufacturer’s instruction.

#### Bacterial 16S rRNA gene sequencing

The 16S rRNA gene Pair-End (PE) amplicon sequencing on V4–V5 regions using the primers 515F (5′-GTGCCAGCMGCCGGCGGTAA-3′) and 1064R (5′-CGACRRCCATGCANCACCT-3′) was performed on the microbiome DNA samples at the University of Minnesota Genomics Center. DNA sequence data for this experiment are available at the NCBI Bioproject repository (BioProject: PRJNA720397). The abundance matrix, metadata and taxonomy are available at Zenodo (10.5281/zenodo.5703837). Please see Supplemental File 1 for more detail.

#### Processing amplicon reads and designating operational taxonomic units (OTUs)

Processing of the 16S rRNA gene PE amplicon sequencing data was done as described in detail [27]. In short, the VSEARCH workflow [28] was used to process and curate the OTU table. A critical aspect of the OTU clustering was setting the stringency 99.5% (id percentage = 0.995). This is roughly equivalent to allowing for 1 nucleotide mismatch. Quality control was run on the OTU table to remove samples with low coverage (less than 10,000 OTUs total). We also excluded OTUs with proportionally low or high coverage. To set these cutoffs, we rescaled the counts for each sample proportionally to 617,753, the size of the sample with the largest number of counts. We then removed OTUs with less than 100 counts (which would represent <0.02% of the data for that sample) or more than 200,000 counts (which would represent more than 33% of the data for that sample). This yielded 92,385 OTUs. To facilitate comparisons across the OTU table and between samples, each OTU count in each sample was scaled proportionally. The taxonomic identity of each OTU was determined using both SILVA (version 138) and RDP 16 S rRNA gene databases augmented with the known 16S rRNA gene sequences of the individual SynCom strains. For all stacked bar plot analyses (Figs. 3b, e, 5b, d and S5) as well as the indicator species analysis (Fig S4), OTUs with ‘unknown’ designations or those that were likely plant plastid derived, were manually removed. The α-diversity metric (Shannon diversity) was calculated using the diversity function from the vegan package [29]. Spatial influence on the microbiome data was evaluated using ANOVA and spatial correction was performed using the method described in ref. [27]. After accounting for these sources of variation, uniform manifold approximation and projection for dimension reduction (UMAP) [30], both unsupervised and supervised approximations, were used to assess the treatment effects on global microbiome profile. In addition, permutation ANOVA was performed on the derived OTU table to assess the significance of treatment, microbe inoculum, and their interaction on the overall composition using the vegan::adonis function. Using 1000 iterations and the Bray–Curtis dissimilarity measurement, the partial correlation and *p* value for each source of variation was recorded (Table 1). The interaction between the drought and microbial treatments was used as the supervision factor (calibrated abundance ~  Drought × SynCom). OTUs with significantly differentiated abundance in each microbial treatment under drought were identified using the indicspecies (indicator species) package in R [31]. The results of the indicator OTUs in different microbial treatment groups were visualized as Venn Diagram using Venny (https://bioinfogp.cnb.csic.es/tools/venny/). Calibrated abundance at the phylum level was fitted with the generalized linear mixed model based on negative binomial distribution (nb glmm) to detect the enrichment. The Lsmeans function in lsmeans package in R was used to test the significance of the enrichment effects in the resulting models. The *p* values were adjusted using the method of Benjamini-Hochberg (FDR was controlled to the level of 0.05).

**Table 1:** PERMANOVA *p* values and partial correlations (corr), iterations = 1000.

Phenotyper			
	**Drought**	**Microbes**	**Interaction**
*p* value	0.001	0.001	0.001
partial corr	0.114	0.056	0.035

Field: *p* values			
	**Treatment**	**Genotype**	**Interaction**
Root	0.001	0.001	0.911
Rhz	0.001	0.001	0.914
Soil	0.001	0.001	0.993

Field: partial corr			
	**Treatment**	**Genotype**	**Interaction**
Root	0.017	0.082	0.062
Rhz	0.040	0.092	0.057
Soil	0.013	0.089	0.053

#### Cluster analysis and heat map to define the indicator OTUs

The relative abundance matrix of the indicator OTUs compared to the control (indicator OTUs × samples) was calculated by dividing the abundance of each indicator OTU in its sample over the median abundance of that indicator OTU in the control samples. A hierarchical clustering was applied over the relative abundance matrix using the function hclust in the stats package in R. The relative abundance matrix of the indicator OTUs was further visualized using a heatmap. The rows in the heatmap were ordered according to the dendrogram order obtained from the cluster analysis of the indicator OTUs. The heatmap was colored on the basis of the log_2_-transformed fold-change compared to the control.

#### Change-point models associating the plant phenotypes and microbiome abundance

Calibrated abundance of each OTU was fitted against both plant phenotypes, plant area and fresh shoot weight, with the change-point model, a function provided by R chngpt package. An OTU was considered a hit if the slope of the line after the estimated threshold is significantly non-zero for both plant phenotypes. The significant OTUs were visualized using upset, a function provided by R UpSetR. For each compartment, the list of significant OTUs was manually examined to determine how many were either both negatively (Table S2) or both positively (Table S3) correlated with area and fresh weight. These numbers are reported as an inset (Fig. 3d).

### Sorghum field experiment (Figs. 4 and 5, Fig. S5)

#### Field layout and experimental design

The field was located near Scottsbluff Nebraska at 41°53′39.4″N 103°41′06.1″W. The previous crop grown in this field was dry bean. Nitrogen (urea) was applied to the field area and incorporated using light tillage at a rate of 90 kg ha^−1^. Plots consisted of four rows, 76 cm apart and 4.6 m long. A split-plot design was implemented using eight replicate blocks for two watering treatments and 24 sorghum genotypes. The two watering treatments were delivered with a variable-rate lateral irrigation system which supplied 31.75 cm of water to the well-watered treatment and 3 cm to the water-stressed treatment. The well-watered plots were irrigated every 7–10 days and the water-stressed plots were initially irrigated to allow the crop to emerge and then irrigation was stopped. Seeds were supplied by the Kresovich, Rooney and Dweikat labs, were treated with Concep III, and were planted on June 7, 2017. Glyphosate at 1.54 kg a.i. ha^−1^ and S-metolachlorat 1.42 kg a.i. ha^−1^ were sprayed to the field one day after planting. Final biomass harvest and sampling was done on September 19, 2017.

#### Field harvest measurements and microbiome sampling

The fresh and dry weights of plots were measured by hand harvesting a 91 cm section of a row. The number of stalks was recorded, panicles were cut off and stalks and panicles, when present, were weighed separately. After weighing a 91 cm section, a subset of stalks and panicles were dried to a constant weight and a dry weight to fresh weight ratio was calculated to determine the dry weight of the entire 91 cm section. Plant heights were measured as the average height of plants in one of the two center rows of the 4-row plot with a telescoping measuring stick which could be aligned with the top of the plants. The plant phenotype data was then normalized based on a soil chemical spatial structure as previously described [27]. In short, various soil properties (pH, sum of cations, base saturation, soluble salts, organic matter, nitrate-nitrogen, phosphorus, potassium, calcium, magnesium, sodium, sulfur, zinc, iron, manganese, and copper) were measured across the field. Each of these properties were then assessed for an effect on plant phenotypes and then further assessed for correlation between the properties. This allowed us to collapse the influence of various soil properties into a limited number of principle components and account for these effects within our models. The plant phenotypes were further normalized by removing the genotype effects after calibration from the soil’s chemical spatial distribution.

#### Field sample collection and DNA extraction

DNA was extracted from roots, rhizosphere and the bulk soil for two plants in each plot using methods described in McPherson et al. [32] and all samples were sent for 16S rRNA gene amplicon sequencing at JGI. Briefly selected plants were excavated using a shovel. The excess soil (approximately 200 g) from the excavated root ball was shaken off and collected into quart-size Ziploc bags. A representative sample of root types from each plant were cut with a scissor and placed in 50 mL tubes with phosphate buffer (6.3 g L^−1^ NaH_2_PO_4_, 8.5 g L^−1^ Na_2_HPO_4_ anhydrous). After vigorous shaking, the roots were removed from the tubes and placed in new 50 mL tubes. The soil that was released from the roots (rhizosphere) was saved in the 50 mL tubes with phosphate buffer. The rhizosphere, roots and soil were placed on ice and transported to the laboratory. Solutions of sodium hypochlorite (5.25%) and ethanol (70%) were used to surface sterilize the roots for 30 s in this respective order, followed by washing three times with sterile ultrapure water. Roots were then cut and frozen in 15 mL tubes. Liquid N was then used to grind the roots to homogenize and access the endosphere microbial communities. The rhizosphere samples were first filtered (100 µm mesh) to remove large debris, then pelleted (3000×*g* for 10 min) and resuspended with 1.5 mL phosphate buffer. After transferring to a sterile 2 mL tube, the rhizosphere was re-pelleted and the supernatant was discarded. The rhizosphere pellet, the ground roots and a small sample of soil were stored in 2 mL tubes at −20 °C until DNA extraction. The remaining soil was stored in the Ziploc bags at 4 °C. The rhizosphere and bulk soil DNA extraction was performed using the MoBio PowerSoil-htp 96-well soil DNA isolation Kit, while the endosphere DNA was extracted using the Applied Biosystems (ThermoFisher Scientific) MagMax Plant DNA isolation kit. A KingFisher robot was used to automate the DNA extractions.

#### Bacterial 16S rRNA gene amplicon sequencing

DNA was quantified and then amplified in 96-well plates with single indexed primers targeting the V4 region of the bacterial 16S rRNA gene [33, 34]. Chloroplast and mitochondrial Peptide Nucleic Acid (PNA) blockers were used to prevent chloroplast and mitochondrial amplification in root endosphere samples [35]. Amplified samples were multiplexed at 184 samples per 2 × 300 bases PE MiSeq (Illumina) sequencing. The 16S microbiome raw sequence data is available for download through the JGI user portal: Author: Daniel Schachtman; Title: “Systems Analysis of the Physiological and Molecular Mechanisms of Sorghum Nitrogen Use Efficiency, Water Use Efficiency and Interactions with the Soil Microbiome”; https://genome.jgi.doe.gov/portal/SysAnaMicrobiome/SysAnaMicrobiome.info.html Data is listed under the following titles: Energy Sorghum Plate 2017_“62–98” itags. Alternatively, all raw data is available for download from the project website: http://shiny.danforthcenter.org/sorghum_systems/.

#### Field microbiome data analysis

Data analysis followed the same methodology described above for the Phenotyping Experiment and as described previously [27]. This includes processing of the 16S rRNA gene raw reads, defining and normalizing OTUs, calculating differentially abundant OTUs and using the change-point models to identify positive and negative associated OTUs. Both UMAP and permutation ANOVA were performed as described above on the soil property adjusted OTU table for each tissue compartment (Table 1 and S4).

### Software and code availability

Segmentation and feature extraction of the images was performed with software written in C++ that is freely available at https://github.com/jberry47/ddpsc_phenotypercv and must be compiled against OpenCV (version >=4.0) with the extra modules: ml, aruco, and ximgproc. Additional dependencies are listed in the documentation with instructions on how to install them. Statistical analyses were performed using R version 3.5.2 [36] with the following packages: NBZIMM v1.0, lsmeans v2.30-0, emmeans v1.4.8, ggtext v0.1.0, uwot v0.1.8, forcats v0.5.0, purrr v0.3.4, readr v1.3.1, tidyr v1.1.1, tidyverse v1.3.0, data.table v1.12.8, tibble v3.0.3, doParallel v1.0.15, iterators v1.0.12, foreach v1.5.0, chngpt v2019.11-26, UpSetR v1.4.0, indicspecies v1.7.9, ggrepel v0.8.2, patchwork v1.0.1 ggsci v2.9, ggpubr v0.4.0, gdata v2.18.0, compositions v1.40-3, robustbase v0.93-5, tensorA v0.36.1, DAtest v2.7.11, vegan v2.5-6, permute v0.9-5, gridExtra v2.3, stringr v1.4.0, lme4 v1.1-23, Matrix v1.2-18, scales v1.1.1, reshape2 v1.4.4, car v3.0-9, carData v3.0-4, factoextra v1.0.7, FactoMineR v2.3, corrplot v0.84, Hmisc v4.3-0, Formula v1.2-3, survival v3.2-3, lattice v0.20-41, ggplot2 v3.3.2, plyr v1.8.6, dplyr v1.0.1, dendextend v1.13.4, ggdendro v0.1.21. R script(s) for all data processing and figure generations can be found at Zenodo (10.5281/zenodo.5703837). For help navigating the supplemental files and Zenodo repository, please see Supplemental File 1. Raw image data will be provided upon request.

## Results

### A synthetic community and specific *Arthrobacter* strains caused root-growth inhibition on sorghum seedlings

**Fig. 1:**
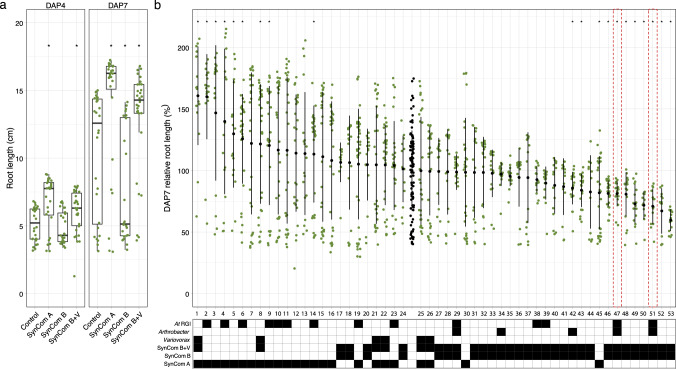
Synthetic communities (SynComs) and individual bacterial strains affect sorghum root length phenotypes in a rapid seedling assay. Green dots represent the root lengths of individual sorghum seedlings. **a** Box plots display medians (horizontal line), the 75th and 25th percentiles (top and bottom of box) and the upper and lower whiskers extend to data no more than 1.5× the interquartile range from the upper edge and lower edge of the box, respectively. **b** Each strain was tested individually (1–53) for effect on sorghum seedling root growth. Additional strain details (Supplemental Table 1). Gray dots represent the control (no bacterial treatment) seedlings. The solid black dots and lines represent the mean ± standard deviation. Specific features of each strain are summarized in the lower indicator table. Black shading indicates that the strain has that feature. RGI: Root Growth Inhibition. Red outline indicates *Arthrobacter* strains (47 and 51) that cause RGI in both *Arabidopsis* and sorghum. The number of replicated samples for each treatment **a**: *n* > 20, **b**: *n* ≥ 11. Wilcoxon rank-sum tests were performed between SynCom treatments and control samples (sorghum without microbial treatments) (**a** and **b**) and *p* values were corrected using the method of Benjamini–Hochberg to control for false discovery rate. *p < 0.05.

Previous work demonstrated that specific synthetic communities cause root growth inhibition (RGI) phenotypes in *Arabidopsis* [20]. To investigate whether the SynComs cause similar phenotypes in sorghum, a sorghum germination assay was performed. Three SynComs were constructed for this assay: SynCom A consisted of 24 strains from a SynCom that did not cause RGI in *Arabidopsis* (Module A in [20]); SynCom B consisted of 29 strains selected from SynComs that did cause RGI in *Arabidopsis* (Modules C + D in [20]); and SynCom B + V consisted of the 29 SynCom B strains plus the six *Variovorax* strains from SynCom A (Table 1). We performed sorghum seedling germination pouch assays to assess the effects of the SynComs on sorghum root development. The results showed that compared to the controls, SynCom A- and B + V-treated sorghum seedlings had longer primary roots, while SynCom B-treated seedlings displayed the shortest roots (Fig. 1a, Fig. S1a). These data suggest that SynCom A and B promote and inhibit sorghum root growth, respectively. Considering the consortium compositions, these results also suggest that *Variovorax* strains in SynCom B + V suppress the RGI phenotype elicited by SynCom B. These results are consistent with what was previously reported for *Arabidopsis* and tomato [20].

Next, we investigated the contribution of individual strains to the RGI phenotype. Each strain was tested using the germination assay (Fig. 1b). At seven DAP, 11 strains (Fig. 1b, Table S1) out of 53 caused RGI in sorghum as compared to the control seedlings, most of which belonged to SynCom B. In addition, this assay revealed that nine SynCom A strains can promote root growth, including two *Variovorax* strains. Out of the 53 strains tested, 14 were previously reported to cause RGI in *Arabidopsis*. Among those, only two strains can also induce short roots in sorghum, both of which were *Arthrobacter* strains (Fig. 1b, Table S1).

Despite statistically significant effects on root growth, we also observed a large amount of variation among experimental replicates. We hypothesized that this may be due to the level and/or location of colonization. Thus, to further investigate bacterial colonization on the sorghum roots we selected two representative strains: *Variovorax* strain CL14, the root growth promoter (the leftmost strain in Fig. 1b), and *Arthrobacter* strain CL28, the root growth suppressor (the third rightmost strain in Fig. 1b) (Fig. S1b). These strains were applied to sorghum seeds and root length was measured at 7 days. In addition, bacterial populations on the root surface and within the root tip were quantified. The results suggest that the *Variovorax* strain CL14 is a robust rhizosphere colonizer (Fig. S1b right). Endosphere colonization of CL14 was only observed in a few of the sorghum roots (green squares) and notably, these replicates showed shorter root lengths compared to rhizosphere colonized roots (green triangles). The control group also had replicates with short roots, suggesting that this short root phenotype may be independent of microbial treatment. *Arthobacter* strain CL28 also colonized the rhizosphere for the majority of replicates, with only a few replicates showing endosphere colonization (Fig. S1b left). Among the replicates with rhizosphere CL28 colonization, we observed a trend towards shorter sorghum root length. This suggests that there may be a dose-dependent effect for the strength of RGI by CL28. Taken together, these data support our hypothesis that the variation observed may be at least partially explained by the location and level of colonization, even in this relatively controlled experimental system.

### Drought and microbial treatments alter sorghum growth in a high-throughput, controlled environment experiment

Based on the data (Fig. 1a), we hypothesized that SynCom B-treated sorghum would show increased susceptibility to abiotic stresses such as drought and that SynCom A-treated sorghum plants would show relative tolerance because of their root length phenotypes. In addition, we wanted to investigate whether the SynCom-mediated phenotypes would transfer to more complex non-sterile environmental conditions. To address these questions, we performed a 25-day-long experiment using the high-throughput Bellwether phenotyping platform (Lemnatec system) and measured the above-ground phenotypic effects on sorghum growth of the SynComs across the well-watered and drought conditions.

**Fig. 2:**
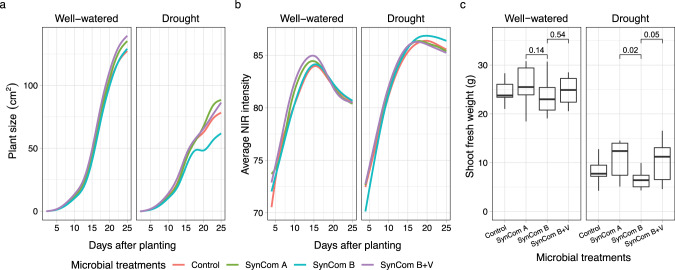
SynComs affect plant growth phenotypes in a high throughput phenotyping assay. The temporal changes of plant size (**a**) and NIR signal (**b**) were plotted using LOESS smoothing, with line colors showing the microbial treatments. **c** The green dots represent the shoot fresh weight of sorghum at the conclusion of the assay. Box plots display medians (horizontal line) the 75th and 25th percentiles (top and bottom of box) and the upper and lower whiskers extend to data no more than 1.5× the interquartile range from the upper edge and lower edge of the box, respectively. Pairwise *t*-tests were performed between microbial treatments for well-watered and drought conditions. The *p* values for select comparisons are shown and all others were not significant (alpha = 0.05). The number of replicated samples for each treatment *n* = 50 (**a** and **b**) or *n* ≥ 10 (**c**).

Sorghum seeds were germinated in the presence of microbes, planted in steam sterilized soil and loaded onto the phenotyping platform. All pots were well-watered for four days prior to starting the drought treatment. Every plant was weighed and then watered if necessary (below target weight) and imaged each day for a total of 25 days. Both RGB and near-infrared (NIR) images were collected. The NIR intensity may be used as a proxy for water content wherein a lower value correlates with higher plant water content [37]. At the end of the experiment, fresh and dry shoot weights were quantified. In our experiment, 80% of the variance in plant area was explained by the treatment factors, meaning plant area robustly responded to the treatments (Fig. S2a, b). Plotting plant size and NIR intensity over time revealed several striking differences (Fig. 2a, b). First, a clear drought treatment effect was observed for the control (no microbial seed treatment) plants as measured by reductions in plant area and increased NIR intensity, and this correlated with fresh and dry shoot biomass at the end of the experiment (Fig. 2c, Fig. S2c). This strong correlation between plant area and biomass is consistent with previous reports [26, 38]. In addition, considering the microbe treatments, we observed that under drought conditions, SynCom A- and SynCom B + V-treated plants performed better than SynCom B-treated plants. These patterns were observed for plant area, NIR intensity and shoot fresh weight at the end of the experiment (Fig. 2). We also considered shoot dry weight and water content at the end of the experiment and while some similar trends were observed, the differences were not significant (Fig. S2c–e).

### *Arthrobacter* and *Variovorax* strains applied to sorghum seeds colonize and persist in sorghum roots

The observed phenotypic differences among the microbial treatments suggests that the microbes in SynCom A and SynCom B + V protect sorghum from drought stress while the microbes in SynCom B exacerbate the negative impacts of drought. To test whether the microbes applied at the beginning of the experiment were able to persist with the sorghum plants and to potentially pinpoint specific microbes within each SynCom with major roles in affecting above-ground phenotypes, we characterized the root microbiomes of each plant using 16S rRNA gene amplicon sequencing.

The VSEARCH workflow was used to cluster 16S rRNA gene amplicon sequences at 99.5% identity into operational taxonomic units (OTUs). A total of 7904 distinct OTUs were observed after quality filtering. Of the 53 individual strains that make up the three SynComs, only five strains had corresponding OTUs that were detected at the end of the experiment. This result demonstrates that not all the SynCom strains persisted through the phenotyping assay. This also showed that the majority of OTUs present at the end of the phenotyping assay originated from the non-sterile environment in which the experiment was performed.

**Fig. 3:**
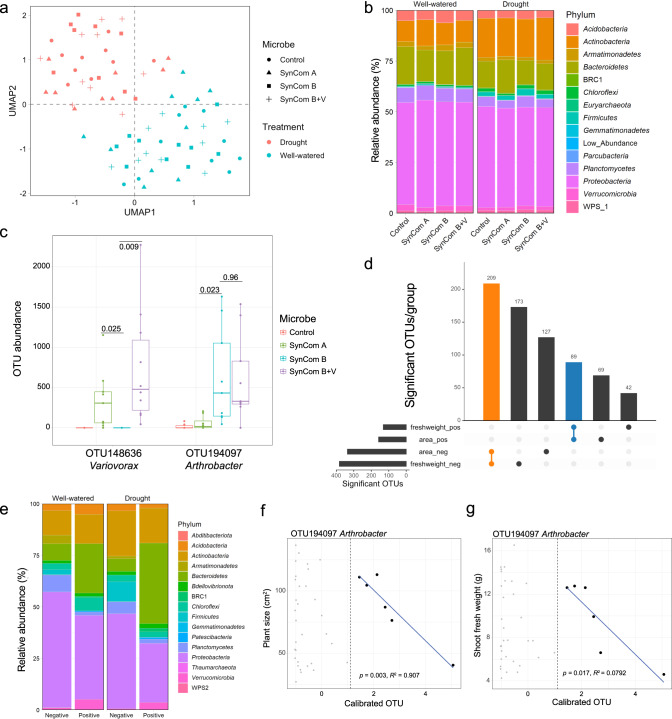
Characterization of the sorghum root-associated microbiome after the high-throughput phenotyping assay. **a** The clustering of microbiome samples using unsupervised UMAP, with colors and shapes showing the drought and microbial treatments, respectively. **b** Phylum-level distribution of the microbial microbiota across treatments. **c** The OTU abundance of *Variovorax* and *Arthrobacter* OTUs at the conclusion of the assay under drought. The dots represent the OTU abundance in different samples with colors showing the microbial treatments. The horizontal bars within boxes represent medians. The tops and bottoms of the boxes represent the 75th and 25th percentiles, respectively. The upper and lower whiskers extend to data no more than 1.5× the interquartile range from the upper edge and lower edge of the box, respectively. Pairwise t-tests were performed between SynCom treatments. The *p* values for select comparisons are shown and all others were not significant at the alpha of 0.05. The numbers of replicated samples *n* ≥ 8. **d** The numbers of OTUs associated to both plant phenotypes. Colors represent the OTU groups with same association directions. **e** Phylum-level distribution of the plant phenotype-associated microbiota within drought treatments. **f**, **g** The change-point model fitting between OTU abundance and plant phenotypes (**f** plant size; **g** shoot fresh weight) for OTU194097 *Arthrobacter* strain. Gray dots indicate samples that did not meet the abundance threshold.

Although the phenotyping experiment was performed within a controlled environment growth chamber, spatial effects may still occur and can introduce unwanted experimental noise. Indeed, although treatments were randomly distributed throughout all four greenhouses, greenhouse 1 (GH1) appeared to have smaller plants than the other greenhouses (Fig. S3a). Therefore, both plant area and the OTU table were adjusted for spatial effects as described (Materials and Methods and [27]). Comparison of the original and calibrated data showed that while plant area did not change significantly, the OTU table did show significant differences (ANOVA, *p* value = 2.2 × 10^−16^) and therefore, the calibrated table was used for subsequent analyses. To assess the effect of drought conditions and microbial treatments on the global microbiome profiles, we considered an unsupervised and a supervised uniform manifold approximation and projection (UMAP) analysis. While a strong drought treatment effect on the microbiome was observed with both approaches (Figs. 3a and S3b), only the supervised UMAP was able to detect a microbe treatment effect on the microbiomes, which is consistent with a strong effect from the environmental (non SynCom-derived) microbiome. To test whether drought influenced the diversity of microbial communities, we considered the Shannon diversity among the different treatment groups. The SynCom A- and B + V-treated samples had significantly lower Shannon diversity compared to that of the SynCom B-treated samples, suggesting the SynCom A and B + V treatments decreased the richness and evenness of the sorghum rhizosphere microbiome (Fig. S3c). Comparing the microbiomes at the phylum level revealed several groups of microbes that were differentially abundant between the treatments (Fig. 3b). For example, *Actinobacteria* (which includes *Arthrobacter*) were more abundant in the drought treated samples (Fig. S3d).

To identify specific OTUs that were enriched among the microbe treatment groups under drought, we employed the ‘indicator species’ algorithm [31]. The resulting lists of OTUs were compared to the SynCom starting inoculums to look for overlap (Fig. S4). For example, SynCom A inoculum comprises 18 unique strains (Fig. S4a). At the end of the experiment, we observed 5 OTUs that were specific to SynCom A-treated plants (Fig. S4b) and indeed, the taxonomic annotations of these 5 OTUs were among the original list of SynCom A strains (Fig. S4c). In this manner, across all treatments, 18 OTUs were identified as likely SynCom-derived OTUs (Fig. S4c). These OTUs represented 1%, 2.2%, and 2.6% of the relative abundance in SynCom A-, B- and B + V-treated plants, respectively. These data indicate that only a subset of the SynCom strains was able to persist throughout the experiment and again highlight the significant environmental component of the microbiome.

Based on recent reports on *Arthrobacter* and *Variovorax* and our sorghum seedling data (Fig. 1), we were particularly interested in OTUs corresponding to these two genera. Under drought, SynCom A and SynCom B + V shared one enriched OTU which corresponded to *Variovorax* (OTU148636) (Fig. 3c, Fig. S4c). SynCom B and SynCom B + V shared 13 enriched OTUs, all of which had matches from the starting inoculums and one of which corresponded to *Arthrobacter* (OTU194097) (Fig. 3c, Fig. S4c). We note that this OTU was also detected in SynCom A/drought treated plants, albeit at a lower abundance possibly suggesting some level of contamination between the treatments or a similar microbe present in the environment. These data suggest that *Arthrobacter* and *Variovorax*, applied to sorghum seeds, were able to persist with developing sorghum roots over the course of the four-week experiment.

Further, these analyses revealed many additional OTUs, presumably from the non-sterile environment in which these experiments were performed, that were specifically enriched or depleted in the presence of specific SynComs. A large group of OTUs was depleted during drought stress and in the presence of SynCom A but only a few of these were also depleted in SynCom B + V suggesting that additional SynCom A strains influence the resulting microbiome (Fig. S4c). These results suggest that not only can many of the SynCom strains persist in a complex environment, they may also dramatically shape the resulting microbiome in a stress responsive manner.

### Colonization by *Arthrobacter* and *Variovorax* strains correlate with increased and reduced sensitivity to drought, respectively

Next, we queried the dataset for OTUs whose abundance (based on read count abundance) correlated with plant phenotypes under drought, regardless of the microbial treatments. We reasoned that a given microbe may only influence plant phenotype once a certain amount of colonization was achieved. Change-point models accommodate this concept by allowing for no effect on the plant phenotype until a certain abundance threshold is reached, after which a linear trend between quantity of a microbe and phenotype is observed. A microbe is considered a “hit” for having a significant impact on a plant phenotype if the regression slope after the estimated threshold is significantly non-zero, either negative or positive. Further, to reduce the amount of false-positive hits we assessed two plant phenotypes, plant area and fresh shoot weight, for every microbe. To qualify as a ‘hit’, the OTU had to exhibit significance in both phenotypes in the same direction.

In total, 209 and 89 OTUs, within the whole OTU table, were negatively and positively associated with both plant phenotypes under drought, respectively (Fig. 3d, Tables S2 and S3). The relative abundance of plant phenotype associated OTUs at the phylum level were distinct between positive and negative associations (Fig. 3e). The OTUs that were positively associated with plant growth were more likely to be *Bacteroidetes* (FDR adjusted t-test after log10 transformation *p* value = 2.16*10^−5^) and less likely to be *Firmicutes* (FDR adjusted *t*-test after log10 transformation *p* value = 2.1*10^−33^) than those negatively associated with plant growth. These results suggest that bacteria within the *Bacteroidetes* and *Firmicutes* phyla may have positive and negative effects on plant growth, respectively.

We cross-referenced the plant phenotype associated OTUs (Fig. 3d) with those that showed differential abundance during drought treatment among the four microbial treatments (Fig. S4b). This yielded eight OTUs, all of which negatively affected plant phenotype and seven of which (all non-SynCom-derived) were depleted in the SynCom A samples under drought. This suggests that SynCom A treatment may decrease the abundance of deleterious environmental strains under drought conditions. Further, the eighth OTU identified from the two lists, OTU194097 (*Arthrobacter*), was among the inoculum strains for SynCom B and B + V and showed a significant negative correlation with plant phenotypes (*p* value = 0.003, *R*^*2*^ = 0.907 for plant area; *p* value = 0.017, *R*^2^ = 0.792 for fresh shoot weight) (Fig. 3f and g). Combining these results with those from the sorghum seedling assay, we conclude that *Arthrobacter* strains are deleterious for sorghum growth under drought stress. Notably, the five *Arthrobacter* strains evaluated in Fig. 1 all cluster into a single OTU—OTU194097.

### *Arthrobacter* strains negatively impact sorghum under drought conditions in the field

**Fig. 4:**
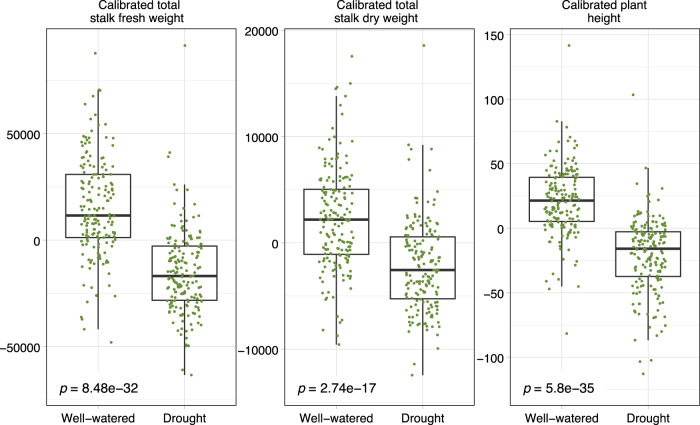
Drought treatment had negative impact on sorghum growth phenotypes in the field assay. The green dots represent the growth phenotypes of the sorghum plant samples after being adjusted for soil property effects. The horizontal bars within boxes represent medians. The tops and bottoms of the boxes represent the 75th and 25th percentiles, respectively. The upper and lower whiskers extend to data no more than 1.5× the interquartile range from the upper edge and lower edge of the box, respectively. *T*-tests were performed between the drought treatments with *p* values shown.

In parallel to the high-throughput phenotyping experiment described above, we performed a large-scale field experiment. In 2017, 24 varieties of sorghum were evaluated for performance across well-watered and drought conditions. Multiple sets of data were collected, including plant traits at the final harvest (plant height, fresh and dry stalk weight, and panicle weight), soil chemical content and properties (calcium, magnesium, phosphate levels, etc.), and microbiome samplings from three below-ground compartments for each plot (root, rhizosphere and soil). Initial analyses revealed strong evidence of heterogeneous spatial distribution of soil factors. Therefore, all plant phenotypes were calibrated to exclude soil nutrient and spatial effects. This approach is described in detail in [27]. In brief, soil nutrients were dimension reduced to the first three principal components and were regressed against using a linear model that included the spatial covariance structure. Calibrated phenotypes were the raw residuals of this model and were used in the subsequent analysis having accounted for these covariates. Further, all the plant phenotypes were adjusted for genotype effects using linear regression and retaining the residuals. After soil factor calibration and genotype adjustment, plant biomass phenotypes, including plant stalk weight and plant height, all suggested that the drought treatments had impaired the plant growth (Fig. 4), which validated the drought treatments.

**Fig. 5:**
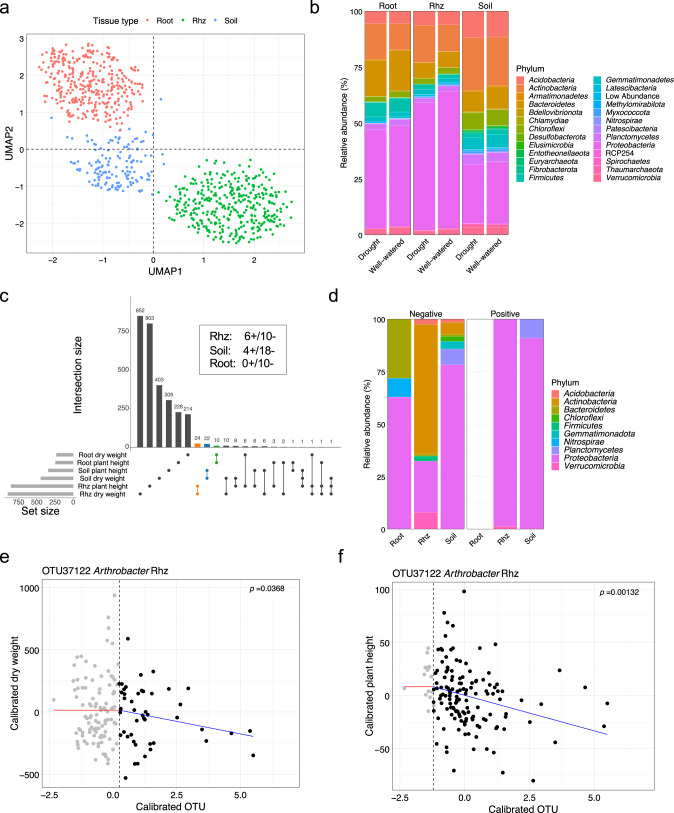
The sorghum microbiome with drought treatments in the field assay. **a** The clustering of microbiome samples using unsupervised UMAP, with colors showing the tissue compartments; **b** Phylum-level distribution of the sorghum microbiota within drought treatments and tissue compartments. **c** The numbers of OTUs associated to both plant phenotypes. Colors represent the OTU groups within the same tissue compartments. Inset box indicates OTUs that display either positive (+) or negative (−) correlation for both samples. **d** Phylum-level distribution of the plant phenotype-associated microbiota within the tissue compartments under drought. **e**, **f** The change-point model fitting between OTU abundance and plant phenotypes (**e** plant dry weight; **f** plant height) for OTU37122 *Arthrobacter* strain. Gray dots indicate samples that did not meet the abundance threshold.

To investigate the microbiome composition associated with each plant, DNA was extracted from three compartment samples (root, rhizosphere (rhz), and soil) and analyzed. After quality control, the OTU table was calibrated to account for the spatial soil property effects [27]. To visualize general relatedness between the sample types, we considered an unsupervised UMAP analysis. The data clustered most strongly by tissue compartment (Fig. 5a). To understand treatment and genotype effect size within each compartment, we ran a PERMAnova (Table 1). Both treatment and genotype had a significant effect on all three compartments (*p* value ≤ 0.001). Effect size was comparable among the three compartments, with rhizosphere showing a slightly higher effect. We also considered a supervised UMAP and observed that the drought treatment effect was most obvious in the rhizosphere samples, consistent with previous reports (Fig. S5a, b) [7, 10]. Also consistent with previous reports, the Shannon diversity of the microbiome was lowest in the root samples. We did not observe a drought impact on Shannon diversity possibly because all genotypes are collapsed in this analysis (Fig. S5c). *Proteobacteria*, *Actinobacteria*, *Bacteroidetes* and *Acidobacteria* were the most abundant phyla in all samples (Fig. 5b). Across all three compartments, the relative abundance of *Actinobacteria* was higher in drought conditions, as compared to well-watered conditions.

We used a zero-inflated negative-binomial generalized linear mixed model (ZINBGLMM) to identify significantly enriched and depleted OTUs between the well-watered and drought conditions for each sample type (FDR was controlled to 0.05) (Fig S5d). The number of differentially abundant OTUs, as a proportion of the total number of OTUs for each compartment, was smallest in the soil samples (Fig. S5d). These observations suggest that the plant roots actively modulate the root and rhizosphere microbiome in response to abiotic stresses such as drought, consistent with previous reports [7, 9, 10]. *Actinobacteria* were enriched under drought in all three compartments (Fig. S5e), and the genus *Arthrobacter* was among the enriched Actinobacterial genera (Fig. S5f).

As a final step, we applied a similar methodology used for the high-throughput phenotyping study described above to identify putative phenotype-associated microbes from the field data. We queried the field data using change-point modeling with two plant phenotypes, plant height and stalk dry weight. Once soil property effects were removed from the OTU table and plant phenotypes, the analysis revealed 22, 24 and 10 phenotype-associated OTUs from soil, rhizosphere and root, respectively (Fig. 5c) and these OTUs were further broken down into positive and negative associations with both phenotypes and taxonomically compared (48 OTUs in total) (Fig. 5d). The OTU37122 corresponding to *Arthrobacter*, was among the negative plant-phenotype-associated OTUs identified from the rhizosphere-derived samples (Fig. 5e and f). This OTU was also enriched under drought conditions (Fig. S5g). While *Variovorax* was not among the OTUs that positively correlate with plant phenotype, this analysis did reveal several other candidate beneficial bacteria. Cross referencing this list with positively associated OTUs from the high-throughput phenotyper experiment revealed several closely related OTUs (OTU2005, OTU53427 and OTU202293) that are annotated as *Nordella oligomobilis* [39], within the *Rhizobiales* Order based on the NCBI 16S rRNA gene database (Fig. S3e, Fig. S5h (left), Table S3, Table S4).

## Discussion

Drought is one of the most serious and unpredictable challenges associated with modern day farming. Exacerbated by the effects of climate change, farmers without easy access to irrigation increasingly experience crop loss from lack of rainfall [40–42]. Beneficial microbes are often touted as a potential method of providing crops with enhanced drought tolerance [43–46]. However, while many candidate beneficial microbes show promise within controlled settings, researchers struggle to translate these candidates to the field [17]. Similarly, while native soils are rich in microbial diversity, it has proven challenging to isolate individual bacteria or consortia that are beneficial when reapplied in a field setting [47, 48]. Here, we describe a three-pronged experimental approach to identifying microbes that affect sorghum drought stress tolerance.

First, we tested synthetic communities (SynComs) of bacteria designed based on interactions with the model plant, *Arabidopsis* [20]. When applied to sorghum roots, the synthetic communities elicited phenotypes very similar to those observed from *Arabidopsis* (Fig. 1a). However, individual strains were less consistent in their effects. That is, while 14 strains caused a short root phenotype on *Arabidopsis* only two of these strains caused a similar root phenotype when applied to sorghum. Further, in our assay, several additional strains from SynCom B caused root growth inhibition on sorghum (Fig. 1b). These results may indicate some amount of host specificity [49] or simply reflect differences in the experimental assays used for *Arabidopsis* and sorghum. Regardless, these assays pointed to *Arthrobacter* and *Variovorax* as being particularly impactful on sorghum roots, similar to what was recently reported for *Arabidopsis* [20].

Next, we tested the SynComs on sorghum over the course of a 25-day high-throughput phenotyping assay. This system was relatively uncontrolled, compared to the seedling assays, and yet robust SynCom dependent phenotypes were observed (Fig. 2). In total, 18 out of 53 SynCom strains were detected at the end of the experiment. Seeds treated with the SynCom that contained *Arthrobacter* but not *Variovorax*, showed increased sensitivity to drought stress and we observed strong negative correlations between *Arthrobacter* abundance and two distinct measures of plant size (Fig. 3f and g). We hypothesize that the observed phenotypes were due to a root developmental defect caused by *Arthrobacter* and/or other deleterious strains. In the future, we look forward to “in soil” advanced root imaging capability that will provide additional insight into root development during interaction with various microbes and abiotic stresses [50]. For the SynCom strains that were not detected at the end of the experiment, we cannot rule out the possibility that these strains had an early impact on development. While both *Arthrobacter* and *Variovorax* persisted with sorghum throughout the course of the experiment (Fig. 3c) they were each a relatively small fraction of the total bacterial communities. It is possible that these strains impact plant growth, even as a relatively low percentage of the overall bacterial microbe. However, we also note that a large group of non-SynCom strains were depleted in samples treated with SynCom A, suggesting that this treatment had a larger effect on the overall microbiome of these samples, and this may have also impacted plant phenotypes. Most notably, OTU157779 belongs to the genus *Burkholderia*, a known pathogen of sorghum, was depleted in SynCom A and SynCom B + V samples and the abundance of this OTU was negatively correlated with plant health (Fig. S4).

In parallel, we undertook a large field experiment wherein drought stress was applied to various sorghum genotypes. We reasoned that because sorghum is a drought-tolerant plant, we would be most likely to isolate microbes that promote drought tolerance from drought treated sorghum, as has been broadly proposed [51, 52]. Our initial attempts at analyzing field phenotyping and 16S rRNA gene sequencing data from the field revealed an abundance of experimental noise, typical of field experiments. However, after accounting for this variability within our models [27] and applying the change-point model to look for OTUs that show positive or negative associations with the measured plant phenotypes (Fig. S5h), we observed that *Arthrobacter* was among the identified negatively associated OTUs in the rhizosphere (Fig. 5e and f). This result was particularly significant as it suggests that analysis methods are able to reveal biologically relevant patterns. While we did not observe significant correlations for OTUs that correspond to *Variovorax* in any analyses for this field, we did identify 10 OTUs that showed positive correlation with plant phenotypes including three OTUs that fall in the Order *Rhizobiales*, which includes previously described beneficial and pathogenic microbes [53].

When cross referencing the plant growth associated OTU lists from the phenotyping and field assays, we noticed a common ‘hit,’ most similar to the previously described *Nordella oligomobilis*, that is positively correlated with plant phenotypes. Little is known about this type of bacteria; however, it falls within the Order *Rhizobiales* [39] along with multiple OTUs corresponding to genera within Family *Bradyrhizobiaceae*. This Family of *Alphaproteobacteria*, especially the Genus *Bradyrhizobium*, includes many slow-growing symbiotic rhizobial strains, many of which are beneficial for their host plants by forming nitrogen-fixing nodules. Recent studies suggested that many non-symbiotic *Bradyrhizobium* species are ecologically important for the soil microbiota and such ecotypes dominate the coniferous forest soil [54–56]. Moreover, *Bradyrhizobium* strains were reported previously to degrade auxin [57–59], whose level plays an important role in plant resilience [20]. Thus, it is tempting to speculate that these newly observed beneficial *Bradyrhizobiaceae* strains may have plant growth promoting properties including auxin degradation, which complement the absence of *Variovorax* in this soil.

Six common genera were negatively associated with plant phenotypes under drought in both assays (field and phenotyping assay), including *Arthrobacter*, *Marmoricola*, *Noviherbaspirillum*, *Paenibacillus*, *Pseudolabrys* and *Pseudomonas*. *Arthrobacter*, *Marmoricola* and *Paenibacillus* are gram-positive genera but have not been extensively characterized [60, 61]. Consistent with previous studies [7, 9, 10], we observed an enrichment of *Actinobacteria* under drought stress (Figs. S3d and S5e) and further presented evidence that *Arthrobacter* strains suppressed plant growth, especially under drought stress (Figs. 3f and g, 5e and f). It is well known that *Pseudomonas* strains have diverse effects on plant growth, including plant growth promotion from *Pseudomonas putida* and *Pseudomonas fluorescens* strains and plant diseases caused by pathogenic *Pseudomonas syringae* strains [62–65]. In general, our results suggest that under drought stress, *Pseudomonas* is detrimental to sorghum growth. Other than *Pseudomonas*, the remaining genera are relatively understudied [66, 67] and so future work will focus on culturing these bacteria and then investigating these strains for potential mechanisms of plant growth suppression. Having cyro-preserved a portion of each field derived root sample, our next endeavor will be to isolate and test these specific candidates.

## Conclusions

We have demonstrated that specific isolates of *Arthrobacter and Variovorax* that affect dicot root growth also affect root growth in sorghum, a monocot. Through a three-pronged approach that spanned sterile, controlled environment and field experiments, we have identified a high confidence list of novel candidate beneficial microbes. This systems-level approach allowed us to mitigate significant environmental noise to reveal underlying robust biological interactions.

## Supplementary information

Supplemental File 1

Supplemental Figures

Table S1

Table S2

Table S3

Table S4
