# Correction: Identification of beneficial and detrimental bacteria impacting sorghum responses to drought using multi-scale and multi-system microbiome comparisons

**DOI:** 10.1038/s41396-023-01442-9

**Published:** 2023-06-15

**Authors:** Mingsheng Qi, Jeffrey C. Berry, Kira M. Veley, Lily O’Connor, Omri M. Finkel, Isai Salas-González, Molly Kuhs, Julietta Jupe, Emily Holcomb, Tijana Glavina del Rio, Cody Creech, Peng Liu, Susannah G. Tringe, Jeffery L. Dangl, Daniel P. Schachtman, Rebecca S. Bart

**Affiliations:** 1 Donald Danforth Plant Science Center, St. Louis, MO USA; 2 Washington University, St. Louis, MO USA; 3 Department of Biology, University of North Carolina at Chapel Hill, Chapel Hill, NC USA; 4 Howard Hughes Medical Institute, University of North Carolina at Chapel Hill, Chapel Hill, NC USA; 5 Curriculum in Bioinformatics and Computational Biology, University of North Carolina at Chapel Hill, Chapel Hill, NC USA; 6 DOE Joint Genome Institute, Lawrence Berkeley National Laboratory, Berkeley, CA USA; 7 Department of Agronomy and Horticulture, University of Nebraska-Lincoln, Lincoln, NE USA; 8 Department of Statistics, Iowa State University, Ames, IA USA; 9 Environmental Genomics and Systems Biology Division, Lawrence Berkeley National Laboratory, Berkeley, CA USA; 10 Carolina Center for Genome Sciences, University of North Carolina at Chapel Hill, Chapel Hill, NC USA; 11 Curriculum in Genetics and Molecular Biology, University of North Carolina at Chapel Hill, Chapel Hill, NC USA; 12 Department of Microbiology and Immunology, University of North Carolina at Chapel Hill, Chapel Hill, NC USA; 13 Center for Plant Science Innovation, University of Nebraska – Lincoln, Lincoln, NE USA; 14 Present Address: Department of Plant and Environmental Sciences, Institute of Life Science, The Hebrew University of Jerusalem, Jerusalem, Israel

**Keywords:** Plant sciences, Microbiome, Bacterial host response

Correction to: *The ISME Journal* 10.1038/s41396-022-01245-4, published online 6 May 2022

In this article the author name Kira M. Veley was incorrectly written as Kira W. Veley.

The original article has been corrected.

