# Supplementary material for: Identification of beneficial and detrimental bacteria impacting sorghum responses to drought using multi-scale and multi-system microbiome comparisons
Source: ISME J. 2022 May 6;16(8):1957–69. doi: 10.1038/s41396-022-01245-4 (PMC9296637; doi:10.1038/s41396-022-01245-4)
Supplement: Supplementary file 1 — Supplemental File 1 [file 41396_2022_1245_MOESM1_ESM.docx]

**Supplement File 1. README**

This manuscript describes extensive raw data and code used to produce the reported results. All data and code has been made available through Zenodo (https://doi.org/10.5281/zenodo.5703837). To assist the reader in accessing/navigating the supplemental and Zenodo files, we have included the following list of file descriptions and file paths.

**Supplemental files:**

Table S1: Descriptions of the previously reported SynCom strains (18,20,25) used in this study.

Table S2: List of OTUs and taxonomic designations that showed negative correlations with both plant phenotypes from the phenotyper run (Figure 3d)

Table S3: List of OTUs and taxonomic designations that showed positive correlations with both plant phenotypes from the phenotyper run (Figure 3d)

Table S4: List of OTUs and taxonomic designations that showed negative or positive correlations with both plant phenotypes from the field experiment (Figure 5c)

**Zenodo files:**

Here are the paths to the raw files. For each directory in zenodo, refer to the R files within for more details on how to recreate figures and explanations for files.

MICROBIOME data:

- FASTA: bart_redo.nonchimeras_ee05-100-095.fas
  - This file contains the sequence of each OTU.
- TAXONOMY: fig_PhenoMicrobiome.tar.gz/allPheno_bothDB_clean.csv
  - This file contains the taxonomy designations for each OUT.
- OTU TABLE: Bart_redo.nonchimeras_OTU_table_ee05-100-095.txt.gz
  - This table contains the raw OTU counts from VSEARCH.

POUCH ROOT LENGTH data:

- Fig 1: fig_pouch.tar.gz/rootlengthclean4.csv; fig_pouch.tar.gz/rootlengthclean7.csv; fig_pouch.tar.gz/alldeckrelative.csv
- Fig S1B: fig_pouch.tar.gz/365_402_compsite.xlsx

PHENOTYPER PLANT data:

- Figure 2A (shapes): fig_phenoplant.tar.gz/Pheno_merged_rmoutlier/6390f6d14506d5a04c189eeb05979fb3-shapes.tsv
- Fig 2B (NIR):

fig_phenoplant.tar.gz/Pheno_merged_rmoutlier/6390f6d14506d5a04c189eeb05979fb3-nir.tsv

- Fig 2C, Fig. S2C, D, E (biomass):

fig_phenoplant.tar.gz/pheno3cleanweightFresh_Dry.csv

PHENOTYPER MICROBIOME data

- Fig. 3, S3 (Callibrated OTU table):

fig_PhenoMicrobiome.tar.gz/20201117OTUnew/pheno3_spatial_calibrated_OTUnew_noshannon_longformat.csv

- Fig. S4: fig_PhenoMicrobiome.tar.gz/field2017_Pheno3_sub_v_20200317.csv

FIELD PLANT DATA

- Fig 4 (Biomass):

fig_FieldMicrobiome.tar.gz/20201109/drought2017_allPlantPhenotypes_rawAndCalibrated_PC123_092120.csv

FIELD MICROBIOME data:

- Fig 5 (Calibrated OTU table):

fig_FieldMicrobiome.tar.gz/20201109/Drought2017_allTissue_joined_cleaned_OTUnew_rmBadSamples_PC123_110620.csv

- Taxonomy: fig_PhenoMicrobiome.tar.gz/allPheno_bothDB_clean.csv
