# Supplementary material for: Identification of beneficial and detrimental bacteria impacting sorghum responses to drought using multi-scale and multi-system microbiome comparisons
Source: ISME J. 2022 May 6;16(8):1957–69. doi: 10.1038/s41396-022-01245-4 (PMC9296637; doi:10.1038/s41396-022-01245-4)
Supplement: Supplementary file 2 — Supplemental Figures [file 41396_2022_1245_MOESM2_ESM.pdf]

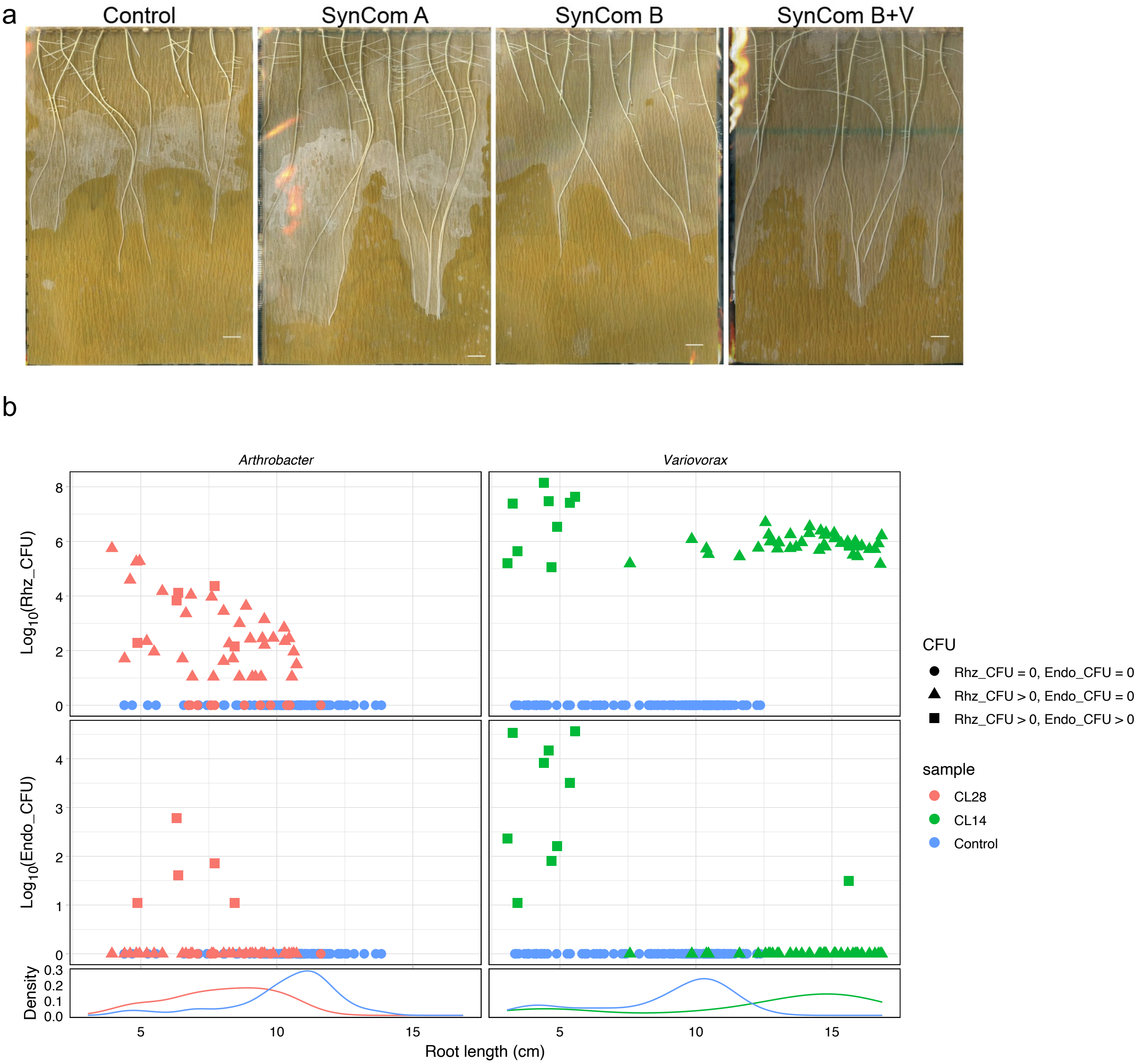

Fig. S1 Sorghum root growth in germination pouches at DAP7 with SynCom (a) and individual strain (b) inoculations. a. Pouch replicate n=3. Bar: 1 cm. b. Colors and shapes represented the bacterial inoculations and colonization tissue compartments, respectively. Rhz = rhizosphere colonization; Endo = endosphere colonization. CL28: *Arthrobacter* strain; CL14: *Variovorax* strain. Control = no microbial treatment. Strain details can be found in Table S1.

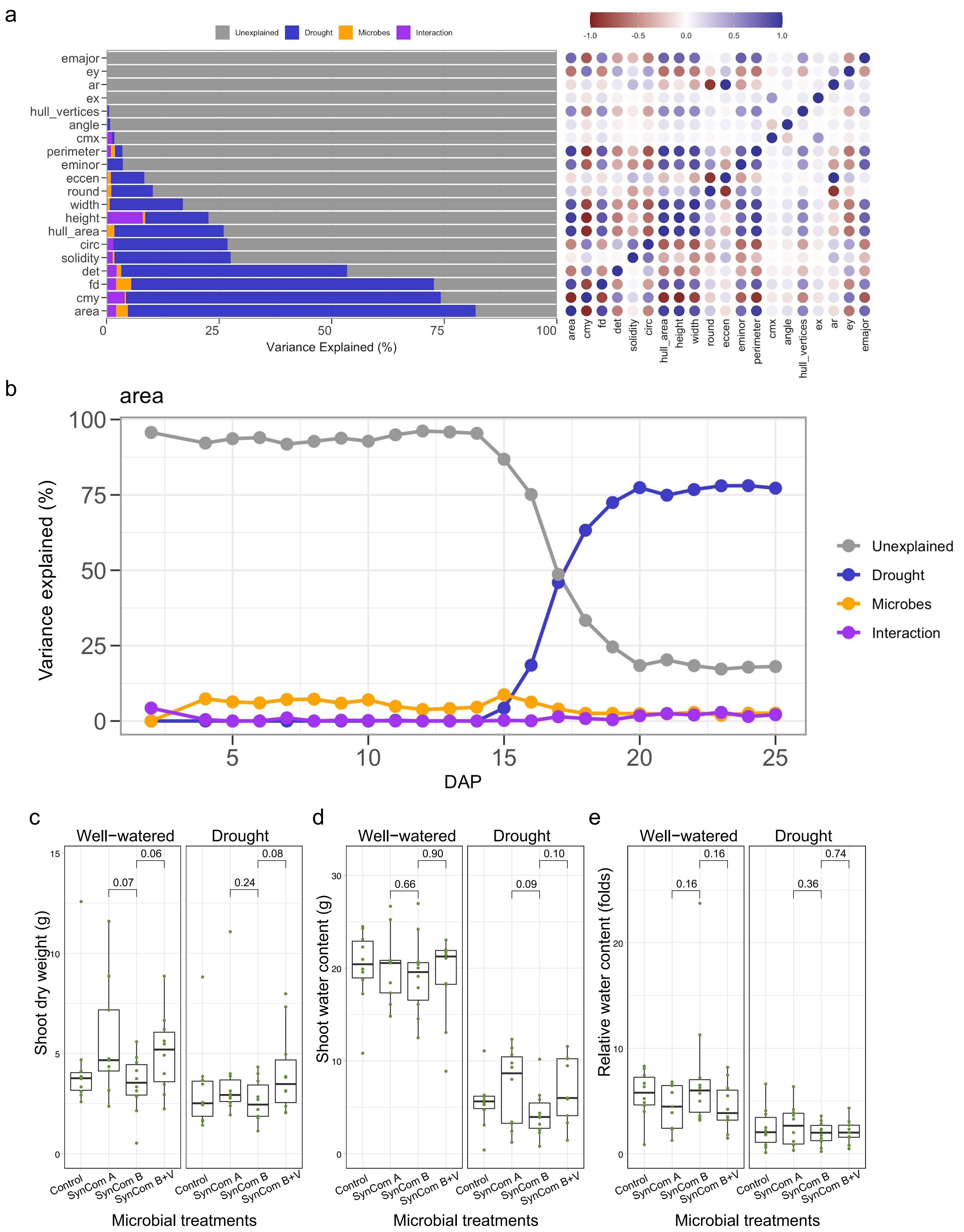

a
Unexplained
Drought
Microbes
Interaction
-1.0
-0.5
0.0
0.5
1.0
emajor
ey
ar
ex
hull_vertices
angle
cmx
perimeter
eminor
eccen
round
width
height
hull_area
circ
solidity
det
fd
cmy
area
0
25
50
75
100
Variance Explained (%)
area
cmy
fd
det
solidity
circ
hull_area
height
width
round
eccen
eminor
perimeter
cmx
angle
hull_vertices
ex
ar
ey
emajor
b
area
100
75
50
25
0
Variance explained (%)
5
10
15
20
25
DAP
Unexplained
Drought
Microbes
Interaction
c
Well−watered
Drought
Shoot dry weight (g)
15
10
5
0
0.06
0.07
0.08
0.24
Control
SynCom A
SynCom B
SynCom B+V
Microbial treatments
d
Well−watered
Drought
Shoot water content (g)
30
20
10
0
0.90
0.66
0.10
0.09
Control
SynCom A
SynCom B
SynCom B+V
Microbial treatments
e
Well−watered
Drought
Relative water content (folds)
20
10
0
0.16
0.16
0.74
0.36
Control
SynCom A
SynCom B
SynCom B+V
Microbial treatments

Fig S2.

Fig. S2 Sorghum growth phenotypes in the high-throughput phenotyping assay. a. PlantCV image analyses pipeline reported variances of 20 plant phenotypes determined with ANOVA (left), and the Pearson correlation matrix among them (right). b. Contributions of different sources of variance on plant area over time. c-e. The green dots represent the shoot dry weight (c), shoot water content (d) and plant relative water content (e) of sorghum at the conclusion of the assay, respectively. Box plots display medians (horizontal line) the 75th and 25th percentiles (top and bottom of box) and the upper and lower whiskers extend to data no more than 1.5× the interquartile range from the upper edge and lower edge of the box, respectively. Pairwise t-tests were performed between microbial treatments for well-watered and drought conditions. The *p* values for select comparisons are shown and all others were not significant at the alpha of 0.05. The number of replicated samples for each treatment n = 10 (c-e).

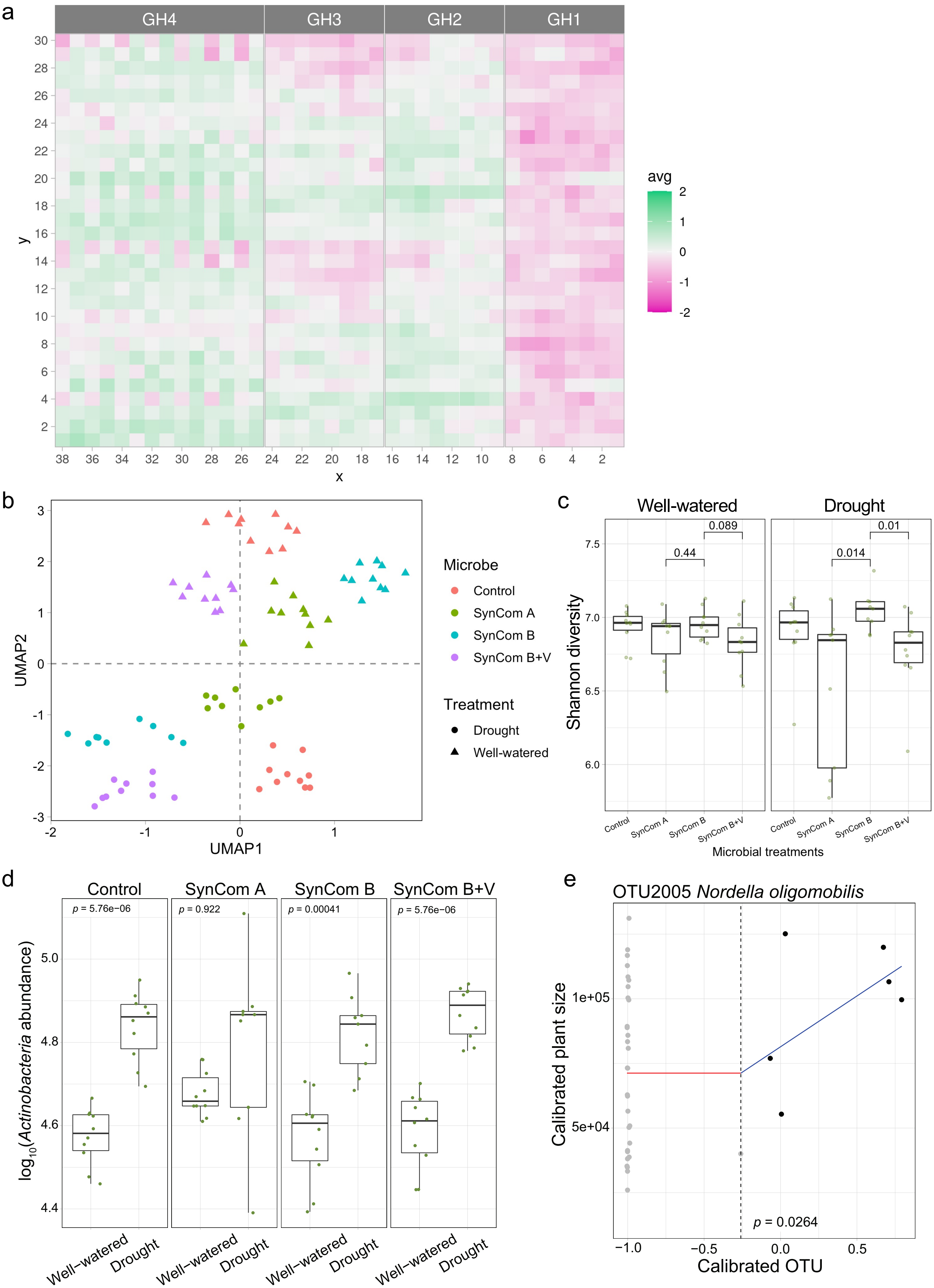
a
GH4
GH3
GH2
GH1
avg
2
1
0
-1
-2
y
x
b
UMAP1
UMAP2
Microbe
Control
SynCom A
SynCom B
SynCom B+V
Treatment
Drought
Well-watered
c
Well-watered
Drought
Shannon diversity
0.44
0.089
0.014
0.01
Control
SynCom A
SynCom B
SynCom B+V
Microbial treatments
d
Control
SynCom A
SynCom B
SynCom B+V
p = 5.76e−06
p = 0.922
p = 0.00041
p = 5.76e−06
log10(Actinobacteria abundance)
Well−watered
Drought
e
OTU2005 Nordella oligomobilis
Calibrated plant size
Calibrated OTU
p = 0.0264

Fig S3.

Fig. S3 Sorghum root-associated microbiome with SynCom and drought treatments in the high-throughput phenotyping assay. a. The spatial distribution of sorghum plant size in the phenotyping facility. GH: greenhouse; avg: z scaled average plant area. b. The clustering of microbiome samples using supervised UMAP, with colors and shapes showing the drought and microbial treatments, respectively. c. The green dots represent the Shannon diversity of sorghum microbiome samples. Pairwise Wilcoxon rank-sum tests were performed between microbial treatments for well-watered and drought conditions. The p values for select comparisons are shown and all others were not significant at the alpha of 0.05. d. The abundance of *Actinobacteria* strains was enriched under drought in three of four microbial treatments. The colors represent the drought treatments. NBGLMM models were fitted between the drought treatments with FDR corrected p values shown. e. Example of OTU abundance positively correlating with plant phenotype based on the change point hinge model. c and d, The horizontal bars within boxes represent medians. The tops and bottoms of the boxes represent the 75th and 25th percentiles, respectively. The upper and lower whiskers extend to data no more than 1.5× the interquartile range from the upper edge and lower edge of the box, respectively. The number of replicated samples for each treatment n = 10.

a

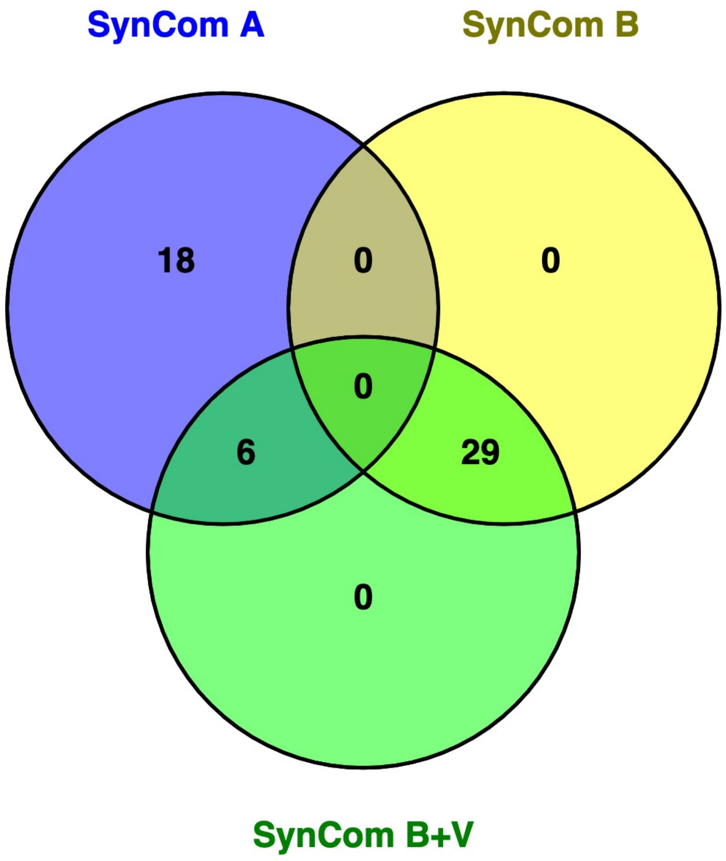

SynCom A
SynCom B
18
0
0
0
6
29
0
SynCom B+V

b

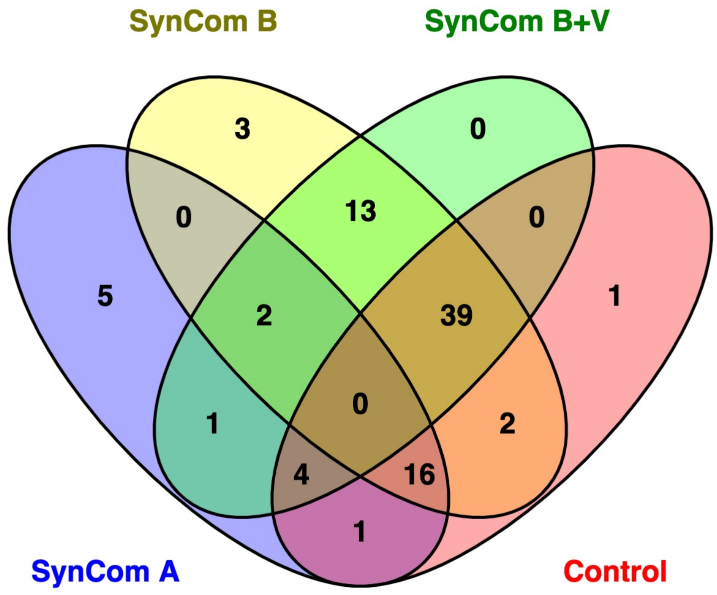

SynCom B
SynCom B+V
3
0
0
13
0
5
2
39
1
1
0
2
4
16
1
SynCom A
Control

c

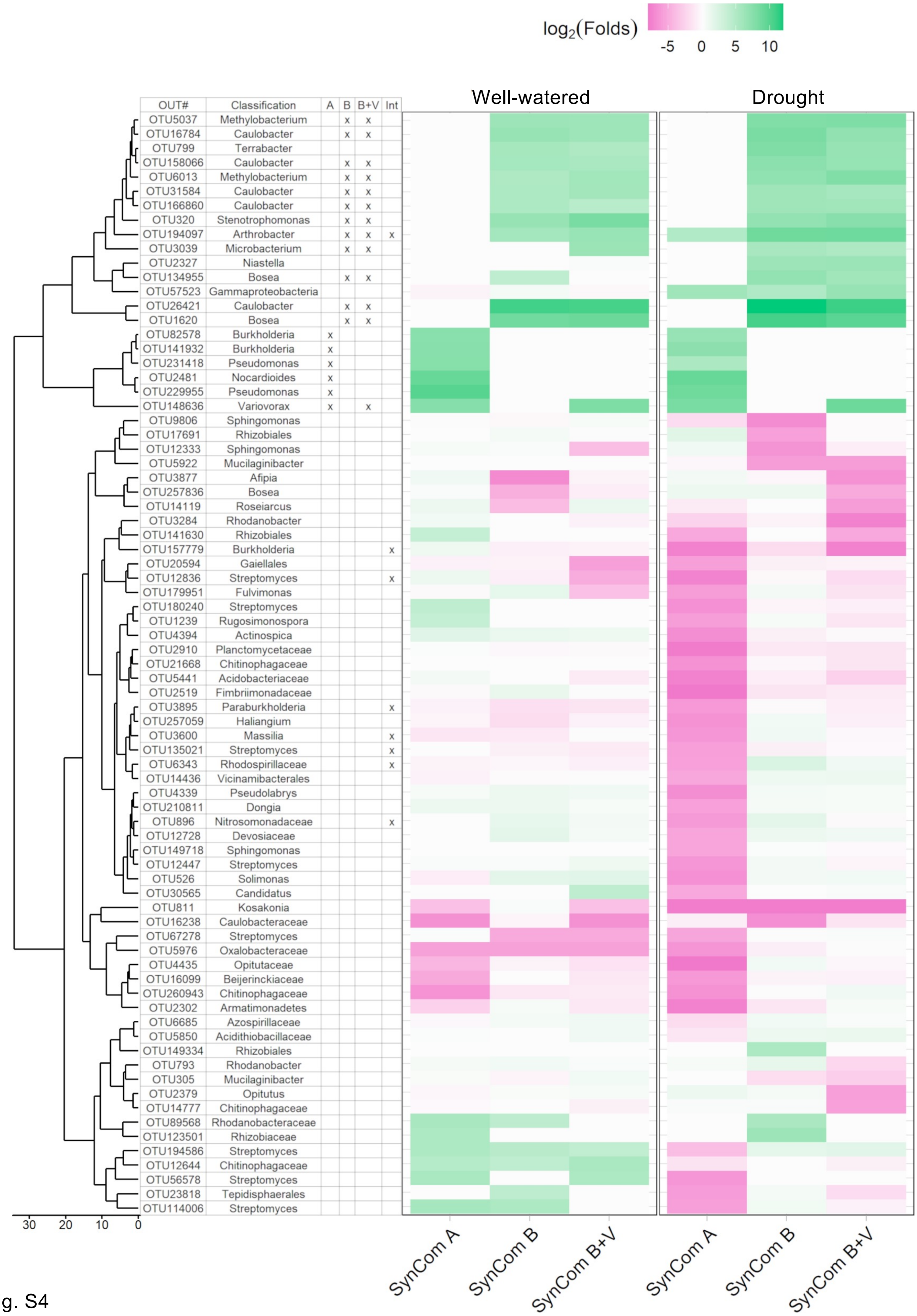
log2(Folds)
-5 0 5 10
Well-watered
Drought
OUT# Classification A B B+V Int
OTU5037 Methylobacterium x x
OTU16784 Caulobacter x x
OTU799 Terrabacter
OTU158066 Caulobacter x x
OTU6013 Methylobacterium x x
OTU31584 Caulobacter x x
OTU166860 Caulobacter x x
OTU320 Stenotrophomonas x x
OTU194097 Arthrobacter x x x
OTU3039 Microbacterium x x
OTU2327 Niastella
OTU134955 Bosea x x
OTU57523 Gammaproteobacteria
OTU26421 Caulobacter x x
OTU1620 Bosea x x
OTU82578 Burkholderia x
OTU141932 Burkholderia x
OTU231418 Pseudomonas x
OTU2481 Nocardioides x
OTU229955 Pseudomonas x
OTU148636 Variovorax x x
OTU9806 Sphingomonas
OTU17691 Rhizobiales
OTU12333 Sphingomonas
OTU5922 Mucilaginibacter
OTU3877 Afipia
OTU257836 Bosea
OTU14119 Roseiarcus
OTU3284 Rhodanobacter
OTU141630 Rhizobiales
OTU157779 Burkholderia x
OTU20594 Gaiellales
OTU12836 Streptomyces x
OTU179951 Fulvimonas
OTU180240 Streptomyces
OTU1239 Rugosimonospora
OTU4394 Actinospica
OTU2910 Planctomycetaceae
OTU21668 Chitinophagaceae
OTU5441 Acidobacteriaceae
OTU2519 Fimbriimonadaceae
OTU3895 Paraburkholderia x
OTU257059 Haliangium
OTU3600 Massilia x
OTU135021 Streptomyces x
OTU6343 Rhodospirillaceae x
OTU14436 Vicinamibacterales
OTU4339 Pseudolabrys
OTU210811 Dongia
OTU896 Nitrosomonadaceae x
OTU12728 Devosiaceae
OTU149718 Sphingomonas
OTU12447 Streptomyces
OTU526 Solimonas
OTU30565 Candidatus
OTU811 Kosakonia
OTU16238 Caulobacteraceae
OTU67278 Streptomyces
OTU5976 Oxalobacteraceae
OTU4435 Opitutaceae
OTU16099 Beijerinckiaceae
OTU260943 Chitinophagaceae
OTU2302 Armatimonadetes
OTU6685 Azospirillaceae
OTU5850 Acidithiobacillaceae
OTU149334 Rhizobiales
OTU793 Rhodanobacter
OTU305 Mucilaginibacter
OTU2379 Opitutus
OTU14777 Chitinophagaceae
OTU89568 Rhodanobacteraceae
OTU123501 Rhizobiaceae
OTU194586 Streptomyces
OTU12644 Chitinophagaceae
OTU56578 Streptomyces
OTU23818 Tepidisphaerales
OTU114006 Streptomyces
30 20 10 0
SynCom A
SynCom B
SynCom B+V
SynCom A
SynCom B
SynCom B+V

Fig. S4

Fig. S4 Analysis of specific OTUs that were differentially abundant across the microbial treatments. The indicator algorithm (31) was used to identify OTUs that were differentially abundant between the samples. a,b. Venn diagrams showing the overlap of strains in the starting inoculums (a) and in roots collected from the drought samples at the end of the experiment (b). c. Relative abundance of microbes identified by the indicator algorithm as differentially abundant. The OTU clusters in the dendrogram (left) were determined according to the OTU abundance profile across the samples. For each OTU, the lowest level classification is listed followed by OTU matches from the initial inoculum (A, B and B+V). The 'Int' column indicates OTUs that were differentially abundant across the treatments and whose abundance correlated (negatively or positively) with plant growth phenotypes. Pink/green heatmap shows relative abundance for each SynCom treated sample compared to the untreated control.

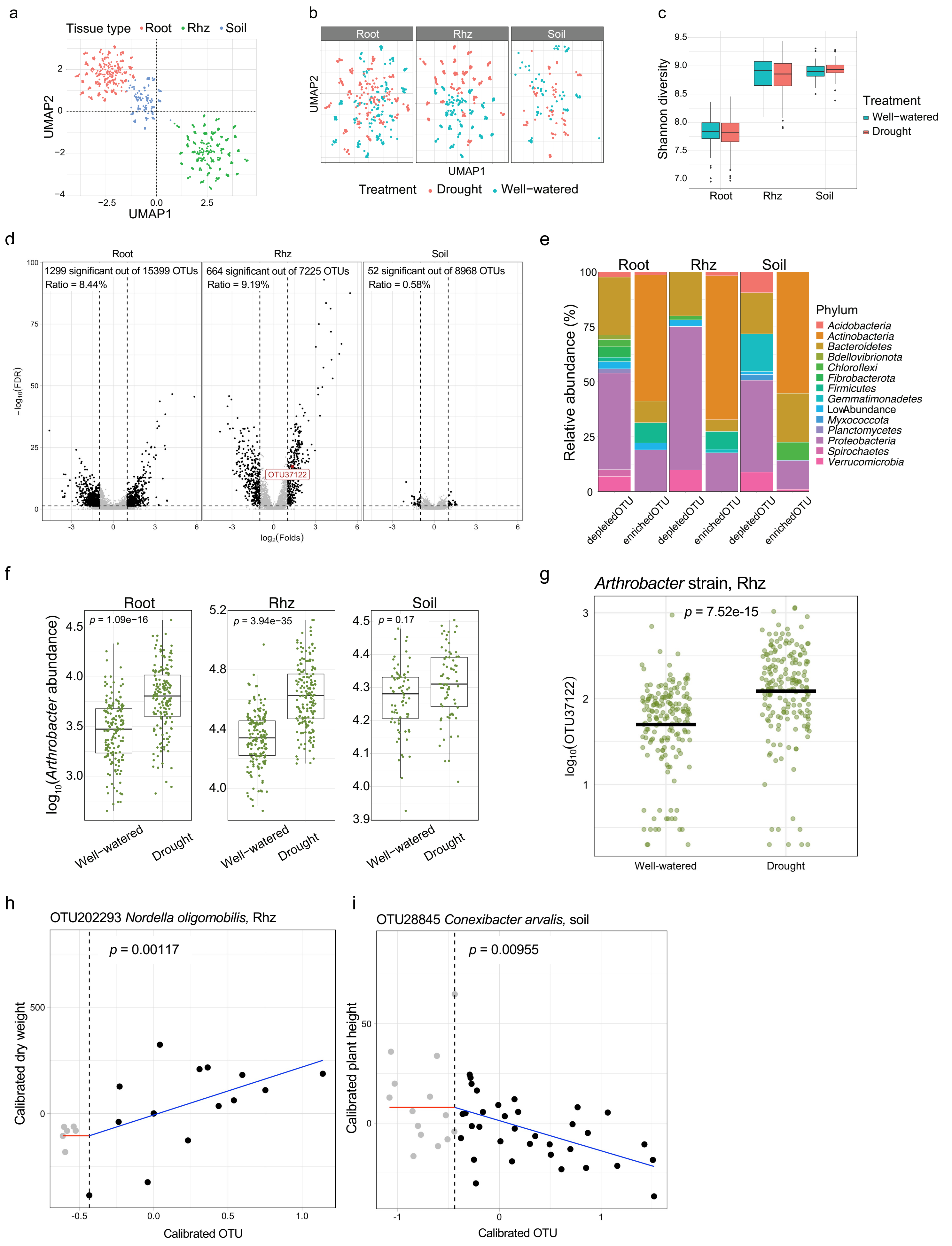
a
Tissue type • Root • Rhz • Soil
UMAP1
UMAP2
b
Root
Rhz
Soil
Treatment • Drought • Well−watered
c
Shannon diversity
Treatment
Well−watered
Drought
d
Root
Rhz
Soil
1299 significant out of 15399 OTUs
Ratio = 8.44%
664 significant out of 7225 OTUs
Ratio = 9.19%
52 significant out of 8968 OTUs
Ratio = 0.58%
OTU37122
−log10(FDR)
log2(Folds)
e
Relative abundance (%)
Phylum
Acidobacteria
Actinobacteria
Bacteroidetes
Bdellovibrionota
Chloroflexi
Fibrobacterota
Firmicutes
Gemmatimonadetes
LowAbundance
Myxococcota
Planctomycetes
Proteobacteria
Spirochaetes
Verrucomicrobia
depletedOTU
enrichedOTU
f
log10(Arthrobacter abundance)
p = 1.09e−16
p = 3.94e−35
p = 0.17
Well-watered
Drought
g
Arthrobacter strain, Rhz
p = 7.52e-15
log10(OTU37122)
h
OTU202293 Nordella oligomobilis, Rhz
p = 0.00117
Calibrated dry weight
Calibrated OTU
i
OTU28845 Conexibacter arvalis, soil
p = 0.00955
Calibrated plant height

Fig. S5 The sorghum microbiome with drought treatments in the field assay. a-b. The clustering of microbiome samples using supervised UMAP, with colors showing the tissue compartments (a) and the drought treatments (b). c. The Shannon diversity of sorghum microbiome samples. The solid dots represent the outliers and the colors show the drought treatments. d. For each tissue compartment (root, rhizosphere (Rhz) and soil), significantly differentially abundant OTUs between well-watered and drought are shown. The dashed lines show the cutoff thresholds: Folds < -2 or folds >2, FDR < 0.05. The dark red label shows OTU37122 *Arthrobacter* strain, which is enriched in Rhz under drought and negatively associated with plant growth phenotypes. e. Phylum-level distribution of the sorghum microbiota with significantly differentiated abundance (from panel d) within the tissue compartments under drought. f. The abundance of *Arthrobacter* strains was enriched under drought in all three tissue compartments. NBGLMM models were fitted between the drought treatments with corrected *p* values shown. g. The abundance of *Arthrobacter* strain OTU37122 was enriched under drought in rhizosphere. The horizontal bars represent medians. ZINBGLMM models were fitted between the drought treatments with corrected *p* values shown. h. Examples of OTU abundance correlating with plant phenotype based on the change point hinge model (OTU202293: Positive association; OTU11030: Negative association). c and f, The horizontal bars within boxes represent medians. The tops and bottoms of the boxes represent the 75th and 25th percentiles, respectively. The upper and lower whiskers extend to data no more than 1.5× the interquartile range from the upper edge and lower edge of the box, respectively.
